# The role of arabinogalactan proteins (AGPs) in fruit ripening—a review

**DOI:** 10.1038/s41438-020-00397-8

**Published:** 2020-11-01

**Authors:** Agata Leszczuk, Panagiotis Kalaitzis, Konstantinos N. Blazakis, Artur Zdunek

**Affiliations:** 1 Institute of Agrophysics, Polish Academy of Sciences, Doświadczalna 4, 20-290 Lublin, Poland; 2 Department of Horticultural Genetics and Biotechnology, Mediterranean Agronomic Institute of Chania, Chania, P.O. Box 85, Chania, 73100 Greece

**Keywords:** Agricultural genetics, Glycoproteins, Agricultural genetics

## Abstract

Arabinogalactan proteins (AGPs) are proteoglycans challenging researchers for decades. However, despite the extremely interesting polydispersity of their structure and essential application potential, studies of AGPs in fruit are limited, and only a few groups deal with this scientific subject. Here, we summarise the results of pioneering studies on AGPs in fruit tissue with their structure, specific localization pattern, stress factors influencing their presence, and a focus on recent advances. We discuss the properties of AGPs, i.e., binding calcium ions, ability to aggregate, adhesive nature, and crosslinking with other cell wall components that may also be implicated in fruit metabolism. The aim of this review is an attempt to associate well-known features and properties of AGPs with their putative roles in fruit ripening. The putative physiological significance of AGPs might provide additional targets of regulation for fruit developmental programme. A comprehensive understanding of the AGP expression, structure, and untypical features may give new information for agronomic, horticulture, and renewable biomaterial applications.

## Introduction

Fruits are one of the main horticultural products. They are eaten fresh or processed delivering pro-health and nutritional components. Therefore, there is a huge demand for growers, retailers, food industry, and consumers for high-quality fruit available throughout the whole year. Although the term “high quality” may mean different properties for each commodity and cultivar, the quality properties of the fruit are formed during its development and ripening, and depend on resistance to stresses, pathogen infections, and physical disintegration of the tissue. Most of the quality parameters of fruit and biotechnological usefulness of plants relate to mechanical characteristics^1^. From mechanical point of view, cells linked with middle lamella, turgor (water content), intercellular spaces and cell wall properties play the key role^2^. Although this macroscopic model of the plant tissue biomechanics is accepted, knowledge about processes at the molecular level that govern cell-to-cell adhesion and remodeling of cell walls is still not sufficient to understand and to control the mechanical properties of the whole fruit. The current cell wall biomechanical model is composed of three polysaccharides: cellulose, hemicellulose, and pectins although the mechanism of how do they assembly in the cell wall is still discussed^3–6^. In general, the cellulose-hemicellulose network performs as a scaffold^7^ while pectins perform as matrix plasticizer in cell walls^8^ and as an adhesive component in middle lamella between cells^9^. This system, especially in the case of pectins, is highly dynamic during fruit development and ripening^10^.

The last two decades research on arabinogalactan proteins (AGPs) since its discovery in fruit tissue^11^ suggests that cell wall assembly may be influenced by these proteins and in consequence, may be also very important for fruit properties. AGPs are cell wall proteoglycans implicated in numerous, variable functions throughout plant growth and development^12–17^. Glycosylation of AGPs constitutes the basis of their essential function and while the molecular mechanisms underlying the function and metabolism of cell wall polysaccharides are well known, the role of AGPs as structural-functional proteoglycans in fruit ripening is described only in several experimental data. The involvement of AGPs in abscission zone differentiation and organ detachment progression is currently under investigation indicating that AGPs might play a regulatory role^18^. Considering that cell wall dissolution is necessary for both fruit softening and abscission zone cell separation to occur it is possible that AGPs might also play a regulatory role in fruit ripening progression. Although the supposed importance of AGP for fruit development and ripening was surely the reason for the rapid increase of published research devoted for fruit in recent years.

The aim of this review is an attempt to associate well-known features and properties of AGPs with their putative roles in fruit ripening. The putative physiological significance of AGPs might provide additional targets of regulation for this developmental programme.

Generally, AGPs are heavily glycosylated hydroxyproline-rich glycoproteins (HRGPs), which consist of up to 95% of carbohydrates. The protein backbone has covalently attached type II arabinogalactan (AG) to polysaccharides made of β-(1,3)-galactan backbones with α-arabinose, β-(1,6)-galactose, β-glucuronic acid, α-rhamnose, and α-fucose. The carbohydrate moiety is characterized by polydispersity due to the different numbers of repetitive AG subunits (1-15)^19,20^. The protein moiety is characterised by the presence of Ala-Pro, Pro-Ala, Thr-Pro, Ser-Pro, Val-Pro, and Gly-Pro peptide repeats. Due to the existence of a glycosylphosphatidylinositol (GPI) anchor on their C-terminus, AGPs are described as GPI-anchored proteins, which are coupled to the outer leaflet of the plasma membrane^21^. The presence of specific phospholipases allows their release into the cell wall and their role as extracellular biosensors. The AGP glycosylation takes place in both the endoplasmic reticulum and the Golgi apparatus. To date, 17 different genes implicated in sugar domain synthesis from the gene family GT31, GT14, GT37, GT29, and GT37 are known. All these genes encode 7 distinct glycosyltransferases corresponding to particular sugar residues of AGPs, e.g., hydroxyproline *O-*β-galactosylotransferase initiating the synthesis process by the addition of the galactose residue onto the hydroxyproline residue core, β-glucuronosyltransferase by the addition of glucuronic acid to galactose chains, and α-fucosyltransferase by the addition of α-fucose residue to AGPs^19–22^.

**Fig. 1:**
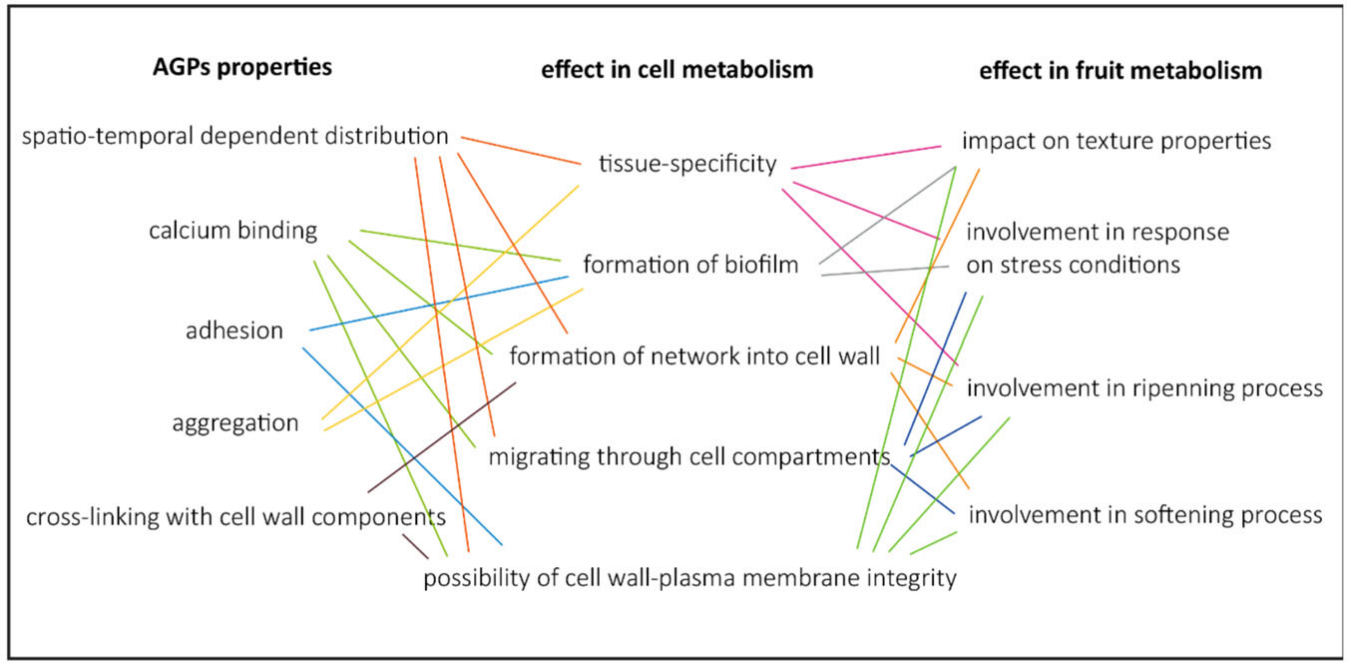
AGPs properties and functions. Mutual correlations between selected AGPs properties, their possible impact on cell metabolism, and involvement in fruit process (fruit metabolism)

Since research on AGPs in fruit is still scarce, possible role for fruit quality might be elucidated to some extent from results obtained for other plant organs. The function of AGPs depends on their precise distribution in cell and their unique chemistry, i.e., a strict structured glycomotif with coupled glucuronic acid residues which bind Ca^2+^,^23^. Genetic manipulation of the protein backbone and changes in glycan moieties by construct transgenic lines shade light on AGP functions. Suppression of prolyl 4 hydroxylases (P4Hs) activity due to silencing of P4H genes results in either lower content or complete absence of AGPs. Lack of proline hydroxylation results in lack of glycosylation of AGPs, leading to alterations in their synthesis and, consequently, their degradation or a shift to lower molecular weight polypeptides^24^. In turn, normal glycosylation has an impact on the proper action of AGPs and thus exerts an effect on cells in many important aspects: ion binding (1), the establishment of cell wall-plasma membrane integrity (2), and cross-linking with other cell wall constituents (3). It is well known that these phenomens occur in plants but are particularly important for fruit quality (Fig. 1).

AGPs are proposed as essential extracellular matrix components that chelate Ca^2+^ by glucuronic carboxyl groups as putative intramolecular Ca^2+^-binding sites. Thus, AGPs are implicated in the Ca^2+^ signaling pathway^25^. There are three presumptions to accept this hypothesis. Firstly, simulations of the molecular dynamics showed that Hyp-AG subunits (repetitive β-(1-3)-linked galactosyl trisaccharide backbone linked β-(1-6) to following galactosyl trisaccharides) contain a putative Ca^2+^, which is the basis for the hypotheses that AGPs are Ca^2+^ capacitors that can be discharged and recharged^20^. Secondly, AGPs and Ca^2+^ situate at the periplasmic area of the plasma membrane, which may comprise significant amounts of Ca^2+^ and acts as a periplasmic reservoir of Ca^2+,^^20^. Thirdly, the mechanism of Ca^2+^ release in the periplasm interface into the cytosol and subsequent recharge is correlated with the abundance of Ca^2+^-binding subunits, free-to-ionize GlcA carboxyls, higher affinity for Ca^2+^ than pectins, higher efficiency of Ca^2+^ binding than pectin, and pH-dependent (at pH 3.2) release of Ca^2+^ from AGPs as a result of H^+^ ATPase activity^25^. The mechanism of these processes is as follows: H^+^ dissociates AGP-Ca^2+^ increasing free cytosolic Ca^2+^, which is involved with the exocytosis of cell wall ingredients^25^. The existence of an AGP-Ca^2+^ capacitor is crucial in AGP functions in morphogenetic patterning and embryogenesis as a primary origin of cytosolic Ca^2+^ waves and, secondly, as a pectic plasticizer^20,25,26^.

Another extremely important property of AGPs is the in vitro and in vivo aggregation tendency^19^ which may suggest this component to be indeed important for cell wall assembly also in fruit. AGPs isolated from a carrot cell suspension showed a strong tendency to self-assemble into larger aggregates composed of smaller ellipsoidal monomers^27^. Similarly, AGPs obtained from transmitting tissues of prepollinated *Nicotiana tabacum* polymerize into various highly ordered oligomer-forming congregations consisting of 10-15 AGP molecules^28^. A high-resolution study of AGPs extracted from *Arabidopsis thaliana* indicates a tendency of AGPs to form clusters, arcs, and branched rings - ‘nanopores’, which is related to situating the carbohydrate moieties outside the AGPs molecule and the protein part inside^29^. Similarly, as a major fraction of gum Arabic, AGPs were described as elongated aggregated structures as an effect of the diversity in the degree of AGP branching and the large size polydispersity of side chains^30^. The first and sole report on AGP fractions extracted from apple juice revealed that removal of arabinofuranosyl substituents caused partial aggregation of AGPs and their enzymatic degradation effects on the haze formation in fruit juices^31^.

These self-associations in agglomerates are consistent with the adhesive ability of AGPs^19^. In *Hedera helix*, AGPs are specified by their ability to agglomerate; hence, it was reasonable to propose aggregation of AGPs within the plant exudate by physicochemical interactions between AGPs. It is well known that calcium ion-driven interactions between AGPs and acidic pectic polysaccharides are the force supporting their cross-linking between the carboxyl groups of uronic acid residues in AGP and pectin. Moreover, the Ca^2+^ driven cross-linking among AGPs and pectic acids supports the consistency of the adjacent AGPs and thus leads to the generation of an adhesive biofilm. In addition, the comparatively low intrinsic viscosity of AGPs is adequate for the wetting activity to create proper cohesive strength^32^. However, the adhesive properties of AGPs exclusively in the fruit have not been investigated to date.

The adhesive properties of AGPs are also essential in establishing a plasma membrane-cell wall connection. AGPs as GPI-anchored proteins are regarded to act in cellular adhesion to maintain the plasma membrane-cell wall continuum^21^. In this context, the adhesive nature of AGPs is related to diverse ways of cross-linking of AGPs with different cell wall components^19^. In charophytes, AGPs were shown to act as key adhesive molecules due to their ability to create cell to cell adhesion and cell to surface adhesion while they were found to be localized in external cell surface and adhesion zones^33^. Interactions in vivo such as covalent glycosidic linkages or non-covalent interactions lead to the participation of AGPs in the cell wall architecture by loosening the pectic network as a pectic plasticizer^34^ and/or by reinforcing the polysaccharide scaffold^35^. The chimeric protein of *Arabidopsis thaliana*—AGP31 interacts in vitro with galactan and rhamnogalacturonan type I (RG-I) through its C-terminal PAC domain (containing the Cys domain). The His-stretch of AGP31 is accountable for the linking with methylesterified polygalacturonic acid by ionic interactions with the carboxyl groups of galacturonosyl residues. The non-covalent interactions demonstrated that the participation of AGPs in the ‘supra-molecular network’ with scaffold-forming cell wall constituents contributed to the ensemble of extracellular matrix^36^. The AGP binding to pectin was examined by Baldwin and co-workers^27^. AGP fractions from red grape wine contain glucuronic acid and galacturonic acid paired with 2- and 2,4-linked rhamnose, indicating the presence of AG-RG fragments^37^. Next, the association between AGPs and pectins was examined in carrot cell cultures, where AGPs were associated to homogalacturonan (HG). It is postulated that the type II AG chain in AGPs is linked to galacturonic acid via ester linkages^38^. Analyses of interactions with pectic components showed that the polysaccharides matrix porosity increases with the upregulation of AGPs by a decrease in pectic cross-linking, which may enhance cell expansion^34^.

Previously, the occurrence of polymeric carbohydrate structures and polyphenol-Hyp-rich glycoprotein complexes were determined in runner bean (*Phaseolus coccineus*), cabbage, and tomato^39,40^. Currently, the most comprehensive study of the connection of AGPs with other cell wall components was performed by Tan and coworkers^41^. In two different glycosylation forms isolated from *Arabidopsis thaliana* culture suspension containing pectin and arabinoxylan glycan domains, covalent binding of carbohydrates to the AGP was recognized. These results showed that AG, RG-I, and arabinoxylan glycan domains were *O*-linked to the Hyp of AGPs. Connections have been identified between RG-I/HG and the rhamnose residue in the AGP type II AG domain as well as arabinoxylan and a rhamnose residue of RG-I or an arabinose residue in the type II AG domain. The complex was named Arabinoxylan Pectin Arabinogalactan Protein1 (APAP1). The carbohydrate domain of APAP1 is formed of both RG-I and HG regions with HG oligosaccharides flanked by RG-I on the pectin backbone. The mentioned conclusions indicate that APAP1 is a proteoglycan that combines an AGP to polysaccharides in the plant extracellular matrix. Results of genetic research indicated that lower expression of the APAP1 protein moiety has effects on the cell wall arrangement. The *apap1* mutation decreased covalent linkages between major cell wall components. Monosaccharide composition studies of the cell wall extracts demonstrated the higher contents of rhamnose, galacturonic acid, and xylose in extracts from mutant walls compared with the wild type. The easier extraction from the *apap1* mutant proves the lack of the core protein in the APAP1 structure. The identification of APAP1 has revealed that AGP is used as a cross-linker for pectin and arabinoxylan, which may be important for the biotechnology of fruit cell walls for preferable agronomic and industrial applications^41^.

Furthermore, in the fruit context, the hormonal regulation of the ripening process has an enormous impact. It is well known about the effect of combined action of phytohormones as major regulators of fruit ripening such as abscisic acid, auxins, gibberellins, cytokinins^42^. Unfortunately, there is a lack of information about the presumed AGPs correlation with hormonal control of fruit ripening. However, according to hormonal regulation of AGP genes expression in other plant tissues, this aspect can be significant also in processes underlying fruit development, maturation, and ripening. Identification of *Arabidopsis* AGP gene allows to show involvement in plant growth, including regulation of germination timing by modulating abscisic acid perception^43^. Furthermore, studies on transgenic lines of tomato (*Solanum lycopersicum*) plants underlined connections between AGPs and auxin-cytokinin signaling and provide the evidence for AGPs role in vegetative and reproductive growth as a transport factor inside the cell^44^. Moreover, inactivation of AGPs function in barley aleurone protoplasts inhibited gibberellin-induced α-amylase synthesis, pointing that AGPs are signaling molecules taking part in the stabilization of a microdomain in gibberellin signal transduction pathway^45^. Similarly, the gibberellin-responsive gene in hypocotyls of cucumber that encodes a protein core specific of AGPs is characterized. Based on the analysis of transgenic plants, AGPs are suspected of implication in hypocotyl elongation which is promoted by gibberellin treatments^46^.

## Discussion

Since today AGPs were examined in a few kinds of fruit, such as goji berry—*Lycium chinese*^11^, *Lycium ruthenicum*^47^, tomato—*Salanum lycopersicum*^24^, grape—*Vitis vinifera*^48^, apple—*Malus domestica*^49–52^, and pear —*Pyrus communis*^53^. Generally, in all these studies, AGPs are characterized as cell wall elements, which are closely related to other polysaccharide constituents with a strong emphasis on their involvement in fruit ripening indicating that particular attention was given mainly on the structural and not the functional features of AGPs.

### Structural characterization of AGPs in fruit

One of the first reports about AGPs isolated from fruit was published two decades ago^11^. AGP was detected in one of the extracted polysaccharide fractions from *Lycium chinense* fruit. This study confirmed extensive glycosylation of AGPs, which are composed of arabinose and galactose at a ratio of 3:1 with a minor content of fucose, xylose, mannose, glucose, and galacturonic acid as well as 3–5% of protein. About 74% of the total amino acid residues were constituted by 5 amino acids, i.e., serine, proline, glutamic acid, glycine, and alanine. A substantial percentage of amino acid residues were bound to carbohydrate, underlining the *O*-glycosidic linkages between both moieties. The analysed fractions contained both branched and linear domains with molecular weight about 20-40 kDa^11^. Further studied on the glycoconjugate isolated from *L. ruthenicum* fruit also consisted of 97.2% of carbohydrate and 1.7% of proteins^47^. This experiment demonstrated that arabinose and galactose were the major sugars with a smaller amount of rhamnose in a molar ratio of 14.9:10.4:1. The extracted sugar chains were polymers composed of a galactan core and side chains with arabinose, galactose, and rhamnose. The Fourier Transform Infrared Spectroscopy (FT-IR) spectrum confirmed characteristic bands for galactose, and the occurrence of peaks at 1250, 950, and 896.7 cm^−1^ indicated a pyranose-type conformation existing in the β-configuration. Furthermore, in the case of *L. ruthenicum*, the AGP protein moiety was represented by hydroxyproline, serine, and alanine. These results show the determination of the AGP structure in *Lycium ruthenicum* as a stronlgy branched molecule with a backbone of (1-3)-linked β-galactopyranosyl residues^47^.

AGPs were also isolated from the juice of apple^31^ and pear^53^. Apple juice AGPs contained arabinose and galactose in a molecular ratio of 0.67 with uronic acid and a protein moiety (1.7%), which form chains of (1-3)-linked galactosyl residues with (1-6)-linked galactan chains and arabinofuranosyl units^31^. Moreover, for apple juice was discovered that removal of arabinofuranosyl substituents by enzymatic dearabinosylation resulted in AGP aggregation, which may be a result of haze formation in apple fruit juices. A few of the polysaccharidic fractions obtained from the pear juice were also characterized as AGPs, thanks to their positive reactivity with Yariv reagent, i.e. formation of precipitate, which is the most specific criterion in the identification of AGPs^54,55^. All analytical data of structural characterization of pear AGPs are coincident with the AGP features discovered earlier: from the glycosidic linkages between galactose and arabinose residues to the low content (2.6%, w/w) of amino acids. Interestingly, successive digestion with α-arabinofuranosidase, exo-β-(1-3)-galactanase and endo-β-(1-3)-galactanase showed that the branched molecule masks β-(1-3)-galactosyl domains in AGPs, i.e., the targeting sites of Yariv reagent^53^.

### Spatio-temporal distribution of AGPs in fruit

The first report about the presence of AGPs during different stages of fruit ripening comes from investigations of grapes fruit^48^. FTIR coupled with Principal Component Analysis and comprehensive microarray polymer profiling using immunological probes showed high amounts of AGPs in ripe fruit samples. The ripening trend was observed in AGPs recognized by LM2 antibody (epitope: (1-6)-β-Gal units with terminal β-GlcA). The proportionally lower abundance of AGPs recognized by JIM13 (epitope: β-GlcA-(1-3)-α-GalA-(1–2)-α-Rha) appeared in green fruit before re-programming ripening started. These findings contributed to identification of a biomarker for grape ripening, whose accumulation is specific to the key phase of grape development and crucial for wine fermentation^48^.

**Fig. 2:**
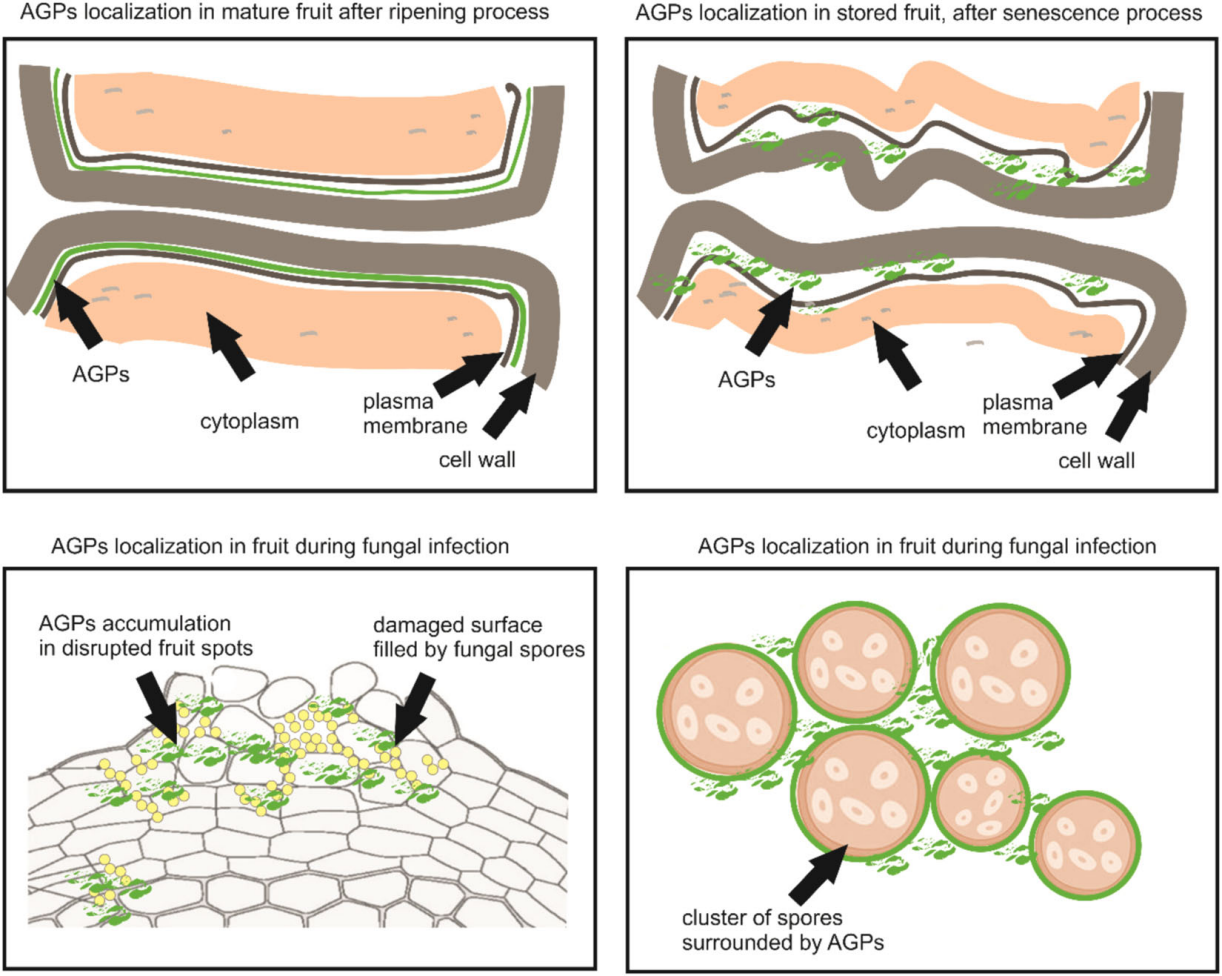
Schematic description of spatio-temporal distribution of AGPs during ripening process and fungal infection The presence of AGPs restricted to the border between cell wall-plasma membrane in mature fruit and AGPs disturbed localization in the whole extracellular matrix after the senescence process. Accumulation of AGPs in damaged fruit zones and spots filled by conidia as a result of substantial disruption of fruit tissue during fungal infection. Surrounding layer of AGPs close to the cluster of fungal spores

The spatial and temporal pattern of distribution in fruit tissue was analyzed in apple^50^ and tomato fruit^17^ in more detail. Immunocytochemical techniques showed that specific epitopes recognizing AGPs were distributed in a greater amount at the edge of vascular bundles, in rings and the spiral lignified thickenings of their walls, as well as inside sclerenchyma cells, mainly in the secondary cell wall of the inner hardened layer of the apple pericarp. Differences in the intensity of immunofluorescence between tissues confirm the tissue specificity of AGPs^50^. Moreover, studies at the cellular and subcellular levels revealed the characteristic presence of apple AGPs in the cell wall-plasma membrane. This is strictly related to the condition of fruit tissue during the ripening and senescence process from the occurrence at the peripheral area of the cell wall and formation of a continuum with the plasma membrane to dispersal over the whole surface of the extracellular matrix after 3 months of postharvest storage. Moreover, the crack of the cell wall-plasma membrane continuum analysed during the fruit softening process was correlated with the remodeling of the AGP arrangement and with a substantial decrease in their epitopes^49^. Similarly, in the case of tomato ripening, borders of the cell wall with the neighboring plasma membrane were full of AGP epitopes^17^. Simultaneous microscopic analyses of AGP and pectins localization in apple fruit showed that de-esterified and methyl-esterified pectins occurred in various parts of the fruit cell, while the absence of AGPs in tricellular junctions excluded the role of AGPs in intercellular adhesion in fruit tissue^51^. Furthermore, the lack of properly formed carbohydrate domains after action with selected cell wall-modifying enzymes showed that the lack of the glycan component had an effect on AGP arrangement by loss or remodeling of AGP epitopes in the fruit cell wall matrix. The absence of glycan chains caused remodeling of the creation of connections between all cell wall components and induced modifications in the whole extracellular matrix^17^. These studies were conducted at different development and ripening stages where substantial decay of fruit firmness occurs and were concluded that the fruit textural properties are the effect of the occurrence of a dynamic matrix connecting polysaccharides and proteoglycans and the synergistic action of these components during the ripening process^17^. The schematic description of AGPs distribution in fruit cells was shown in Fig. 2.

### Identification and gene expression of AGPs in fruit

Based on the variable length and the presence of the signal peptide and conserved domains of AGPs, an effective strategy and searching criteria were used to identify whole gene families. Currently, 325 AGPs are known, including 42 classical AGPs, 9 Lys-rich AGPs, 40 AG- peptides, 5 AGP-extensin hybrids, 98 fasciclin like AGPs (FLAs), 74 phytocyanin-like AGPs, 35 xylogen-like AGPs, and 22 non-classical AGPs. The length of AGP-like sequences and the number of glycomodules have been main variances between classical AGPs, AG-peptides, and chimeric AGPs^55^.

**Fig. 3:**
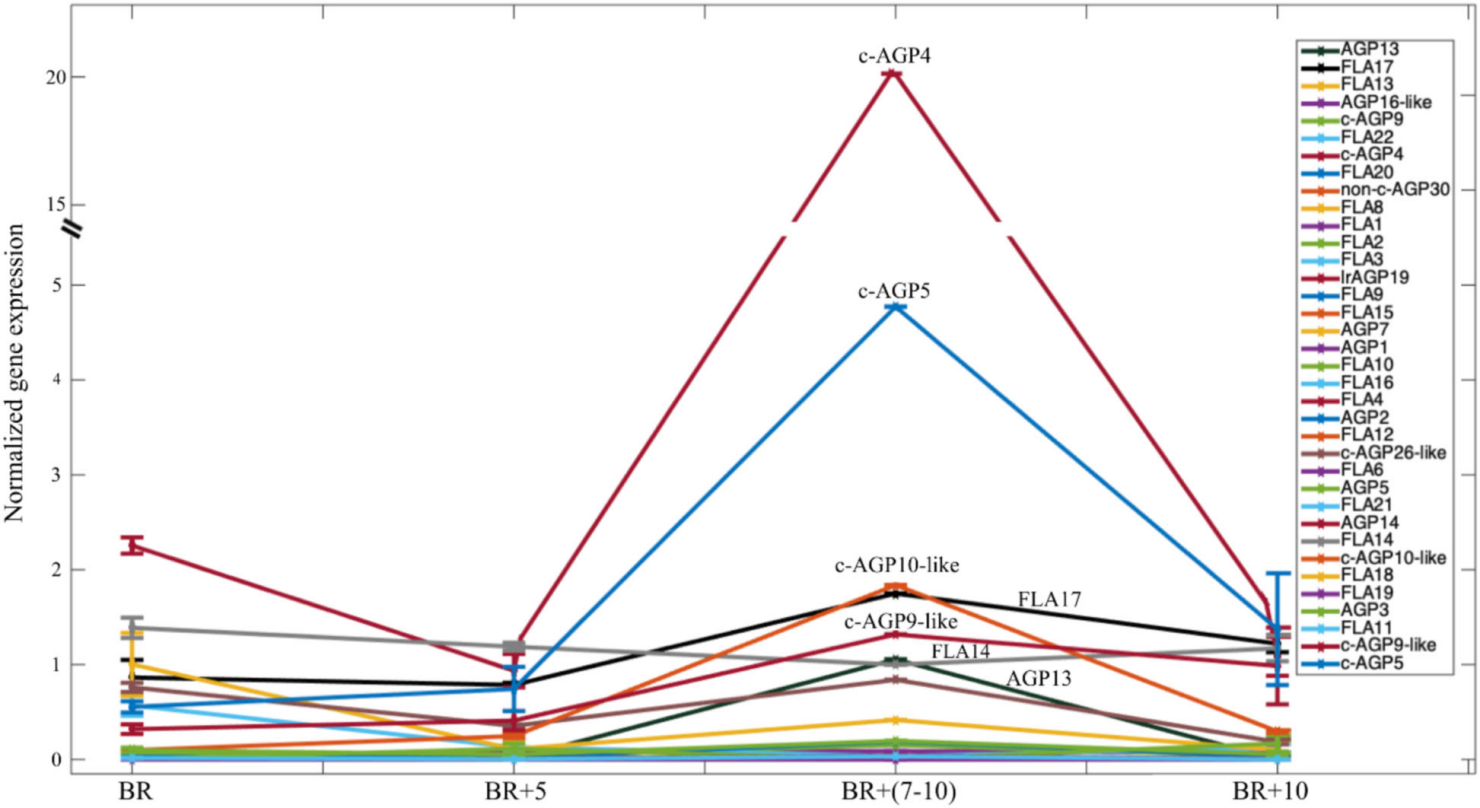
Expression patterns of tomato AGPs in fruit ripening. Normalized gene expression of AGPs expressed in BR (Breaker stage), BR + 5 days, BR + (7–10) days, and BR + 10 days stages of tomato fruit ripening. The BR + 5 days corresponds to either ‘turning’ or ‘pink’ stage, the BR + (7–10) days corresponds to the ‘light red’ and ‘red ripe’ stages and the BR + 10 days corresponds to the ‘red ripe’ stage of ripening. The accession numbers of these AGPs are: AGP1 (Solyc07g064240), AGP2 (Solyc08g066740), AGP3 (Solyc12g013900), AGP5 (Solyc10g011730), AGP7 (Solyc07g053640), AGP13 (Solyc01g005590), AGP14 (Solyc10g078580.1.1), AGP16-like (Solyc01g095520), c-AGP4 (Solyc04g074730), c-AGP5 (Solyc12g057160), c-AGP9 (Solyc01g107340), c-AGP9-like (Solyc12g057140), c-AGP10-like (Solyc11g010390), c-AGP26-like (Solyc09g074450), FLA1 (Solyc06g076110), FLA2 (Solyc07g045440), FLA3 (Solyc07g048090), FLA4 (Solyc08g006300), FLA6 (Solyc10g005960), FLA8 (Solyc06g075220), FLA9 (Solyc07g053530), FLA10 (Solyc07g065540), FLA11 (Solyc12g015690), FLA12 (Solyc09g007660), FLA13 (Solyc01g091530), FLA14 (Solyc10g081720), FLA15 (Solyc07g053540), FLA16 (Solyc08g006290), FLA17 (Solyc01g006820), FLA18 (Solyc11g069250), FLA19 (Solyc12g006110), FLA20 (Solyc05g008320), FLA21 (Solyc10g051090), FLA22 (Solyc03g112880), Lys-rich AGP19 (Solyc07g052680), non-c-AGP30 (Solyc05g052500) genes in the Ailsa Craig cultivar. Data have been retrieved from http://tomexpress.toulouse.inra.fr and presented as mean ± standard error

Initially, 34 putative AGPs were identified in the tomato fruit transcriptome^24^ while after significant improvements of the tomato genome annotation 36 AGPs were identified comprising 7 AGPs, 1 AGP-like, 3 classical AGPs, 3 classical AGP-like, 20 fasciclin like-AGPs, 1 Lysine-rich AGPs and 1 non-classical AGP (Fig. 3). The expression of AGPs genes: SlAGP1 and SlAGP7 changed during the progression of fruit ripening, indicating involvement in this developmental program. A gradual decrease in transcript abundance was observed with the lowest levels at the fully ripe stage of tomato^24^.

The expression of all 36 AGPs was determined during four stages of fruit ripening comprising the Breaker (BR), BR + 5 days, BR + (7–10) days and the BR + 10 days stages by using the TomExpress database (http://tomexpress.toulouse.inra.fr) (Fig. 3). The Breaker + 5 days corresponds to either ‘turning’ or ‘pink’ stage of ripening depending on the conditions of tomato growth such as temperature and solar radiation. The BR + (7–10) days corresponds to the pooling of fruits at the ‘light red’ and ‘red ripe’ stages and the BR + 10 days corresponds to the ‘red ripe’ stage.

The classical AGP4 (c-AGP4) and the classical AGP5 (c-AGP5) exhibited upregulation of expression only at the BR + (7–10) days by ~20- and 5-fold, respectively (Fig. 3). Minor increase in expression at this ripening stage was also observed for classical AGP10-like (c-AGP10-like), FLA17, AGP13, and classical AGP9-like (c-AGP9-like) (Fig. 3). The FLA14 showed slightly higher expression at the breaker stage while it decreased thereafter at the BR + 5 days and BR + (7–10) days. The significance of these AGPs in the ripening process has to be further examined as well as the role of their glycan structure during fruit ripening. Alterations in the frequency of occurrence of AGP’s hydroxylation and possibly glycosylation by either silencing or overexpressing prolyl 4 hydroxylases might alter their structure and subsequently their function leading to changes in fruit growth and ripening. Previously, the silencing of three tomato P4Hs, SlP4H1, SlP4H7, and SlP4H9 by Virus-Induced Gene Silencing (VIGS) resulted in the reduction on the JIM8 AGPs-bound and JIM11 extensin-bound epitopes which might be attributed either to lower levels of AGPs and extensins or to alterations in their glycan structure affecting the binding of the antibodies^52^. Genetic analysis of knock out mutants of Arabidopsis hydroxyproline-galactosyltransferases (Hyp-GALT), which add arabinogalactan polysaccharides in AGPs, showed pleiotropic growth and development phenotypes which were attributed to reduced activity of Hyp-GALT indicating that the AGP glycans are essential for the function of AGPs^22,56^.

The promoter sequences of 7 AGPs, which showed higher expression levels among the 36 AGPs, were identified in silico and the regulatory elements were determined as well as the corresponding binding transcription factors (Table 1).

**Table 1:** Promoter motif analysis of arabinogalactan proteins c-AGP4, c-AGP5, c-AGP10-like, FLA17, AGP13, c-AGP9-like, FLA14

Accession of the regulatory element	Regulatory element	Binding transcription factor	AGP13	cAGP4	cAGP5	cAGP9like	cAGP10like	FLA14	FLA17
AC:RSP00010	G-box	TAF-1	**y**	**y**	*n*	**y**	*n*	*n*	*n*
AC:RSP00039	56/59 box	GT-1 related transcription factors	*n*	**y**	*n*	*n*	*n*	**y**	*n*
AC:RSP00066	Em1b	EmBP-1	**y**	**y**	**y**	**y**	*n*	**y**	**y**
C:RSP000069	G motif	RITA-1; bZIP proteins	**y**	**y**	**y**	**y**	*n*	**y**	**y**
AC:RSP00084	AGL2-BE	AGL2	*n*	*n*	**y**	*n*	*n*	**y**	*n*
AC:RSP00106	AT-1 (cons)	AT-1	**y**	**y**	**y**	**y**	**y**	**y**	**y**
AC:RSP00112	TGA1	Unknown	**y**	*n*	*n*	**y**	**y**	**y**	**y**
AC:RSP00134	G-box	Different bZIP factors, including RITA-1	**y**	**y**	*n*	**y**	*n*	*n*	*n*
AC:RSP00148	GACG-element	Unknown	*n*	**y**	*n*	*n*	*n*	**y**	**y**
AC:RSP00151	CRE, consensus	Unknown	**y**	**y**	**y**	*n*	*n*	*n*	**y**
AC:RSP00169	Element 1	Nodule specific factor	**y**	**y**	*n*	**y**	**y**	*n*	*n*
AC:RSP00173	ElRE (core)	WRKY1; WRKY2; WRKY3	*n*	**y**	*n*	*n*	*n*	**y**	*n*
AC:RSP00174	ATCATC motif	Unknown	*n*	**y**	**y**	*n*	**y**	*n*	**y**
AC:RSP00190	GCN4 box	Unknown	*n*	*n*	*n*	**y**	**y**	*n*	*n*
AC:RSP00228	BOX III	GT-1	*n*	**y**	**y**	*n*	*n*	*n*	*n*
AC:RSP00231	CCAAT box 1	Unknown	*n*	**y**	*n*	**y**	*n*	*n*	*n*
AC:RSP00252	ATMYC BS	rd22BP1 (MYC)	**y**	*n*	**n**	*y*	**y**	*y*	**n**
AC:RSP00282	Amylase-element	Unknown	*n*	**y**	*n*	*n*	**y**	*n*	*n*
AC:RSP00293	PCF2 box	PCF2	**y**	**y**	*n*	*n*	*n*	*n*	*n*
AC:RSP00302	G-box	ABI3	**y**	**y**	*n*	**y**	*n*	**y**	*n*
AC:RSP00326	myc motif	Unknown	**y**	*n*	*n*	**y**	**y**	**y**	*n*
AC:RSP00327	RY	ABI3	**y**	*n*	*n*	*n*	**y**	*n*	*n*
AC:RSP00328	ABRE motif	Unknown	**y**	**y**	**y**	**y**	*n*	**y**	**y**
AC:RSP00359	HVA1s	HvCBF1	**y**	**Y**	**y**	**y**	**y**	**y**	**y**
AC:RSP00363	TATCCAT/C motif	Unknown	*n*	**y**	*n*	*n*	**y**	*n*	*n*
AC:RSP00370	SA/MJ-RE	Unknown	**y**	*n*	*n*	*n*	**y**	**y**	*n*
AC:RSP00390	G-BOX	Unknown	**y**	**y**	*n*	**y**	*n*	*n*	*n*
AC:RSP00416	Box-1	Unknown	*n*	**y**	**y**	*n*	*n*	*n*	*n*
AC:RSP00425	ABRE3a	Unknown	**y**	**y**	**y**	*n*	*n*	**y**	**y**
AC:RSP00427	ABRE4	Unknown	**y**	**y**	**y**	*n*	*n*	**y**	**y**
AC:RSP00437	bZIP-box	Unknown	**y**	*n*	*n*	*n*	**y**	**y**	*n*
AC:RSP00456	E4	DBPF-1; DBPF-2	**y**	**y**	*n*	**y**	*n*	*n*	*n*
AC:RSP00524	E4-core	DPBF-1; DPBF-2	**y**	**y**	**y**	*n*	*n*	**y**	*n*
AC:RSP00553	TSS	Unknown	**y**	**y**	*n*	*n*	*n*	*n*	*n*
AC:RSP00596	box II EE2	Unknown	*n*	**y**	**y**	*n*	*n*	*n*	**y**
AC:RSP00610	UN I2	Unknown	*n*	**y**	*n*	*n*	*n*	*n*	*n*
AC:RSP00629	CCA1 BS2	CCA1	**y**	*n*	*n*	*n*	*n*	*n*	*n*
AC:RSP00643	box 1	TFHP-1	**y**	**y**	*n*	**y**	*n*	*n*	*n*
AC:RSP00646	C1-box	Unknown	*n*	**y**	**y**	*n*	*n*	*n*	*n*
AC:RSP00649	C4-box	Unknown	*n*	*n*	*n*	**y**	*n*	*n*	*n*
AC:RSP00657	G-box	PG1	**y**	**y**	*n*	**y**	*n*	*n*	*n*
AC:RSP00658	G-box (ext)	PG1	**y**	**y**	*n*	**y**	*n*	*n*	*n*
AC:RSP00662	PRD motif 2	Nuclear extract protein(s)	**y**	*n*	*n*	*n*	**y**	*n*	*n*
AC:RSP00664	C-box	Opaque-2 (O2); TGA1	*n*	*n*	*n*	*n*	**y**	*n*	*n*
AC:RSP00666	GCN4 motif	RISBZ1	*n*	*n*	*n*	**y**	**y**	*n*	*n*
AC:RSP00675	Box I	Aleurone layers nuclear protein extracts	*n*	**y**	*n*	*n*	**y**	*n*	*n*
AC:RSP00687	N-box	Unknown	*n*	*n*	*n*	*n*	**y**	*n*	*n*

The promoter sequences of the seven arabinogalactan proteins were identified in the Sol Genomics and the occurrence of functional motifs was described using the ScanWM-PL software (http://www.softberry.com/berry.phtml). Around 50 motifs were determined which were occurred across the seven AGPs. The Table denotes starting from left to right: the accession of the regulatory element, the name of the regulatory element, the binding Transcription Factor and the seven arabinogalactan proteins. The index “**y**” indicates presence of the motif for arabinogalactan proteins, whereas the “*n*” denotes the absence of the corresponding motif. The indexes have been shown in bold if the motif is present “**y**” and italics if it is absent “*n*”

The promoter regions of cAGP4, cAGP5, and AGP13 contain the E4 cis-acting element which was identified in the E4 gene and is well known to be regulated by ripening^57^. This element is considered necessary but cannot confer responsiveness to ethylene on its own^58^. The presence of this motif in the three AGP genes might indicate similar to E4 gene ripening regulation (Table 1).

Four copies of the G-box (CACGTG) cis acting element was identified in the promoter sequences of cAGP4, classical AGP9, and AGP13 indicating contribution in the transcriptional regulation of these genes (Table 1). The G-box motif is bound by the large TF superfamilies of basic helix-loop-helix (bHLH) and basic Leu zipper (bZIP)^59^. Expression of two tomato bZIP TFs, SlbZIP1, and SlbZIP2, under the control of the E8 fruit specific promoter were shown to increase the sugar content in tomato fruits^60^, while three tomato SlbHLH genes were associated with fruit ripening^61^ and an atypical bHLH TF, SlPRE2, affected plant morphology and pigment accumulation^62^. Moreover, the HVA1 regulatory element, which was present in all 7 AGPs, is bound by the *Hordeum vulgare* HvCBF1 TF which was shown to be implicated in the regulation of drought and cold response^63^. The in silico analysis of the promoter region of 7 upregulated AGPs resulted in the identification of *cis*-acting elements bound by specific TFs involved in tomato fruit ripening regulation.

### Effect of modified expression of prolyl 4 hydroxylases (P4Hs) on the AGPs function in fruit

Hydroxyproline-rich glycoproteins, including AGPs, undergo the posttranslational modification of proline hydroxylation catalyzed by P4Hs which is the first step in the glycan synthesis process followed by subsequent rounds of glycosylation with arabinose and galactose allowing AGPs cross-linking into the cell wall to form a covalent network^64^.

Suppression of tomato P4Hs expression leads to putative alterations in the AGPs resulting in abnormal cell division and expansion. The silencing of tomato P4H genes by Virus-Induced Gene Silencing (VIGS) was associated with a reduction in the AGPs content, and a decrease in the shoots and roots mass indicating the impact of this mechanism in biomass production. The downregulation of P4Hs possibly alters the frequency of occurrence of proline hydroxylation in highly hydroxylated AGPs leading to either degradation, structural changes of the glycan part or alterations in their content affecting cell expansion by disarrangement of microtubule organization and the distribution of other cell wall constituents^65^.

The pleiotropic character of P4Hs mutations reveals also the functional importance of AGPs with respect to growth and development. Genetic mutant analysis of tomato P4Hs transgenic lines indicates the importance of the Agp’s content and possibly their glycan chains for the differentiation and development of fruit abscission fracture plane as well as the progression of the natural abscission process^18^. For the first time in tomato, alterations in the function of post-translational modification such as proline hydroxylation revealed the significance of AGPs glycosylation in the differentiation of fruit abscission zone^18^. Microscopic studies of abscission zones showed anatomical alterations in mutant fruit, i.e. variations in the numbers of parenchymal layers indicating cells detained in an undifferentiated stage as well as disruption in lignin deposition^18^.

Moreover, in the stable independent transgenic lines with suppressed expression of *Solanum lycopersicum* Prolyl 4 Hydroxylase 3 - SlP4H3, abscission specific cell wall hydrolases, i.e., polygalacturonase and cellulases were downregulated leading to the prolongation of overripe fruit abscission^18^. These data imply that the manipulation of post-translational alterations might induce changes in the transcriptional activation of a significant number of genes indicating putative regulation of transcription factors and/or involvement of regulatory AGPs.

### Stress factors determining the presence of AGPs in fruit

Plants have evolved numerous strategies to avoid both biotic and abiotic stresses. One of these plant defences are biochemical and structural changes in cell walls including temporal and spatial up- and downregulation of AGPs as a response to low or high temperature, flooding, hypoxia, drought, anoxia, salinity stress, toxicity, mineral deficiency, and microbial infections^15^. In the case of fruit, the molecular mechanisms underlying the multifaceted AGP-dependent reaction are still not well known, except a few reports on gene expressions and AGP occurrence as a response to changeable oxygen conditions, mechanical wounding, and fungal disease.

Modifications on proline hydroxylation induced plethora of changes at the gene expression level. In mammals, the hypoxia-inducible factor, named HIF-1α is a regulator of hypoxic reaction—the stability of which depends on proline hydroxylation by three P4Hs^66^. However, the role of plant P4Hs in intracellular oxygen tension and in the formation of hydroxyproline in nascent transcription factor polypeptides or others of different functions is still unknown.

Proline hydroxylation in the modification of hypoxic and anoxic responses in *Arabidopsis thaliana* seedlings was the subject of a study carried out by Vlad and co-workers^67^. The study demonstrated different patterns of AtP4Hs expression in roots and shoots of seedlings in response to hypoxia and anoxia while 22 HRGPs were identified which were differentially expressed in response to hypoxia. Moreover, the comparatively higher transcript level of AtP4Hs in roots compared to shoots under hypoxia and anoxia treatment was connected with different mechanisms of hypoxic adaptation^67^.

Oxygen deficiency conditions induced different expression of AGPs in tomato fruit indicating possible involvement in stress adaptations. The gene expression of LeAGP1, SlAGP2, and SlAGP4 decreased during the hypoxic time course while after anoxic treatment, the SlAGP4 transcript levels were upregulated 5-fold after 12 h of anoxia. The effect of hypoxia and anoxia on AGPs was also associated with the effect on the regulation of AGP biosynthesis by P4Hs^24^.

In *Arabidopsis* seedlings, the expression levels of 6 AtP4Hs were upregulated in response to mechanical wounding within 6 h of leaf tissue blade wounding^67^. The mechanical wounding response at the molecular level in tomato fruit revealed slight and rapid upregulation of JIM8- and JIM13-bound AGPs within 30 min, which was constant during the experimental time. Interestingly, these AGPs disappeared after 12 h, in contrast to the untreated sample. The relative expression profiles of LeAGP1, SlAGP2, SlAGP4 transcripts were rapidly induced after fruit excision, which is in accordance with the functional importance of AGPs in wound response^24^.

For the first time, the specific distribution of AGPs as a response to fungal infection during postharvest storage was described for apple fruit^52^. This preliminary study focused on the spatio-temporal pattern of the distribution of AGPs in infection-associated modifications in the fruit cell wall and showed the effect of the Yariv reagent during *Penicillium spinulosum* infection. Immunofluorescence technique indicated that JIM13 and LM2 epitopes were increased in the ruptured external tissue layers during the development of the fungal disease. Single *P. spinulosum* conidia were surrounded by AGPs. An increase in AGP epitopes may be correlated with an established impermeable mechanical barrier preventing pathogens from infection of fruit. AGPs may also be obligatory for the creation of infectious structures and affect the fungal infection progress by enclosing the spores in infection zones^52^ (Fig. 2).

## Conclusions

Although the knowledge of the presence and functions of AGPs in fruit tissue is very limited, the available structural and genetic mutant data show that the occurrence of AGPs and in vitro properties may be related to their high importance for fruit metabolism and quality. The cell wall influences the textural features of fruit such as softening during ripening and postharvest storage, which is directly correlated with the distribution of polysaccharides in particular cell wall compartments as well as ripening-related cell wall alterations, i.e., the depolymerization of matrix carbohydrates^68,69^. Some of the AGP functions mentioned above, e.g. their role as cross-linkers in the extracellular matrix are a result of pH-dependent calcium ion binding by AGPs. Considering the significant upregulation of at least two AGPs in tomato fruit ripening (Fig. 3) and the function of AGPs as cross-linkers to pectin polysaccharides it is possible that alterations in the content and/or structure of AGPs might alter their association to pectin and arabinoxylan glycan domains and affect their accessibility to pectin methylesterases and polygalacturonases affecting fruit cell wall dissolution and subsequently softening.

However, the interactions of AGPs with other cell wall constituents, their aggregation, and adhesion abilities, the correlation with the AGP-Ca^2+^ complex in fruit tissue have not been investigated until now. For this reason, basic knowledge of AGPs was helpful for the elucidation of their role in the fruit cell wall-plasma membrane. In summary, obtained up today results give strong evidence that AGPs have participated in cell wall assembly and cell-plasma membrane interaction that means AGPs are also involved in tissue biomechanics. However, this hypothesis is not fully reviewed yet and indeed furthermore comprehensive research on the implication of AGPs in cell wall assembly is indispensable.
